# Predictive Value of Free Triiodothyronine to Free Thyroxine Ratio in Euthyroid Patients With Myocardial Infarction With Nonobstructive Coronary Arteries

**DOI:** 10.3389/fendo.2021.708216

**Published:** 2021-07-28

**Authors:** Side Gao, Wenjian Ma, Sizhuang Huang, Xuze Lin, Mengyue Yu

**Affiliations:** Department of Cardiology, Fuwai Hospital, National Center for Cardiovascular Diseases, Chinese Academy of Medical Sciences and Peking Union Medical College, Beijing, China

**Keywords:** thyroid function, FT3/FT4 ratio, myocardial infarction with nonobstructive coronary arteries (MINOCA), cardiovascular outcomes (CV outcomes), euthyroid

## Abstract

**Background:**

Thyroid function is closely involved in cardiovascular diseases. The free triiodothyronine (fT3) to free thyroxine (fT4) ratio has been reported as a risk factor for coronary artery disease, but its prognostic value in euthyroid patients with myocardial infarction with nonobstructive coronary arteries (MINOCA) remains unclear.

**Methods:**

A total of 1162 euthyroid patients with MINOCA were enrolled and divided according to decreased tertiles of fT3/fT4 ratio. The study endpoint was major adverse cardiovascular events (MACE), including all-cause death, nonfatal MI, nonfatal stroke, revascularization, and hospitalization for unstable angina or heart failure. Kaplan-Meier, Cox regression, and receiver-operating characteristic analyses were performed.

**Results:**

Patients with lower fT3/fT4 tertile levels had a significantly higher incidence of MACE (10.0%, 13.9%, 18.2%; p=0.005) over the median follow-up of 41.7 months. The risk of MACE increased with the decreasing fT3/fT4 tertiles even after multivariate adjustment (tertile1 as reference, tertile2: HR 1.58, 95% CI: 1.05-2.39, p=0.030; tertile3: HR 2.06, 95% CI: 1.17-3.11, p=0.006). Lower level of fT3/fT4 ratio remained a robust predictor of MACE in overall (HR 1.64, 95% CI: 1.18-2.29, p=0.003) and in subgroups. When adding fT3/fT4 ratio [area under the curve (AUC) 0.61] into the thrombolysis in myocardial infarction (TIMI) risk score (AUC 0.69), the combined model (AUC 0.74) yielded a significant improvement in discrimination for MACE (ΔAUC 0.05, p=0.023).

**Conclusions:**

Low level of fT3/fT4 ratio was strongly associated with a poor prognosis in euthyroid patients with MINOCA. Routine assessment of fT3/fT4 ratio may facilitate risk stratification in this specific population.

## Introduction

Acute myocardial infarction (AMI) remains the leading cause of high morbidity and mortality of cardiovascular (CV) diseases worldwide (1). Recently, a distinct population with myocardial infarction with nonobstructive coronary arteries (MINOCA) has been increasingly recognized due to the widespread use of coronary angiography. MINOCA occurs in 5% to 10% of all AMIs and they are younger and more often women compared to those with AMI and obstructive coronary artery disease (CAD) (2–5). It has been found that the prognosis of MINOCA is not trivial and these patients are still at considerable risks for long-term adverse CV events despite optimal secondary prevention treatments (6–10). Thus, it is of necessity to find potential residual risk factors and improve prognosis for MINOCA population.

Thyroid hormones (TH) have been linked with a variety of CV processes. Subclinical or overt thyroid diseases such as hyperthyroidism and hypothyroidism are significantly associated with the development of atherosclerosis and subsequent worse CV outcomes, and the underlying mechanisms may include inflammation, endothelial injury, changes in blood pressure, dyslipidemia, atherogenesis, and cardiac dysfunction (11, 12). Even in euthyroid individuals, minor alterations in TH concentration may lead to increased CV morbidity and mortality (13, 14). Recently, the ratio of free triiodothyronine (fT3) to free thyroxine (fT4) has been suggested as an indirect index reflecting the conversion of T4 to T3 and the peripheral deiodinase activity (15, 16). As reported, the reduction of fT3/fT4 ratio is commonly seen in CV diseases, especially during acute illness (17–19). Meanwhile, lower fT3/fT4 ratio is closely related to unfavorable prognosis in different cohorts with CAD (20–22). However, the predictive value of fT3/fT4 ratio in euthyroid patients with MINOCA remains unclear. Here, we investigated the association between fT3/fT4 ratio and long-term outcomes after MINOCA and explored whether this ratio might provide significant prognostic information in this population.

## Methods

### Study Population

**Figure 1:**
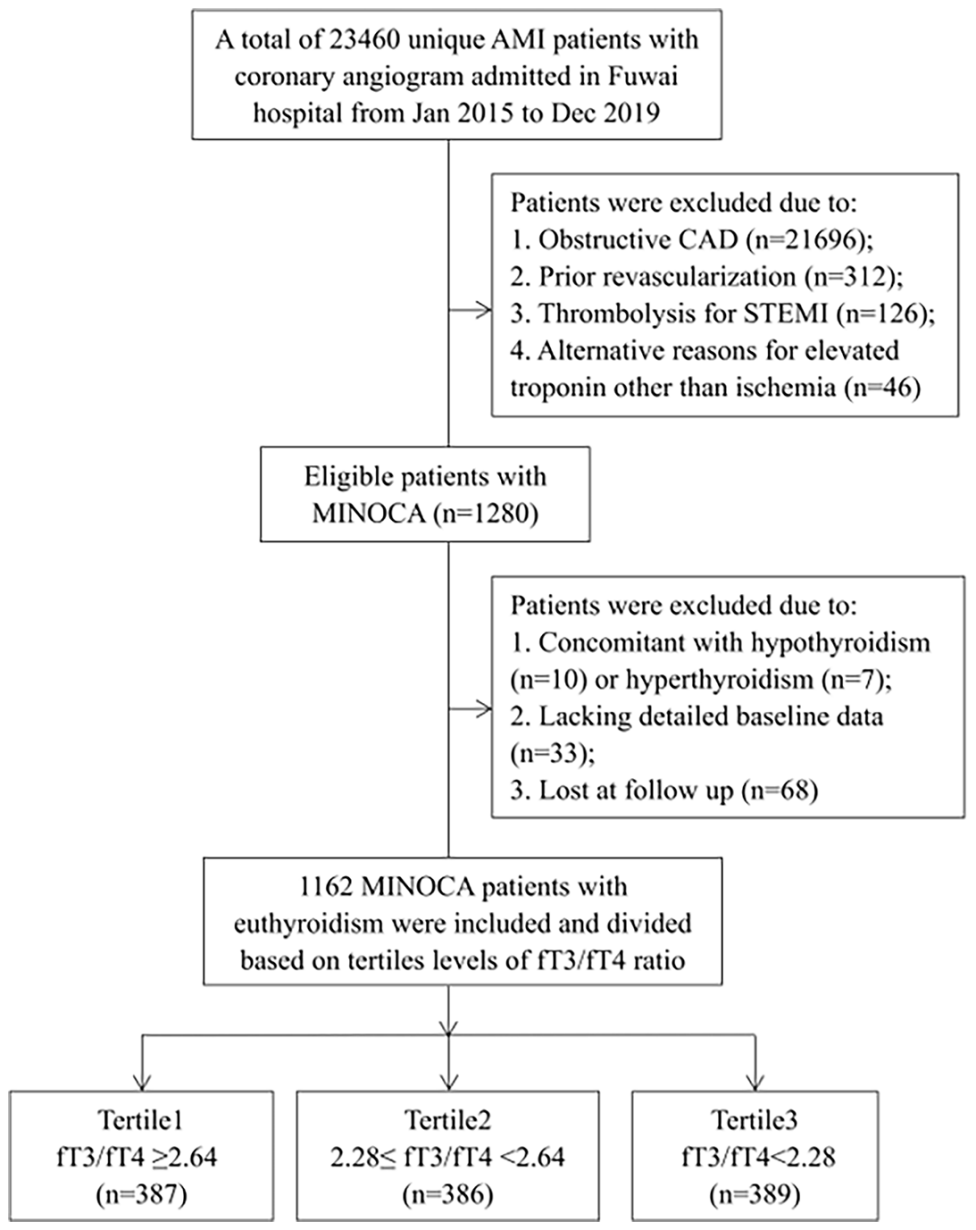
Study flowchart.

This was a single-center, prospective and observational cohort study of patients with MINOCA. From January 2015 to December 2019, a total of 23460 unique AMI patients with coronary angiogram were consecutively hospitalized in Fuwai hospital, including non ST-segment elevation myocardial infarction (NSTEMI) and ST-segment elevation myocardial infarction (STEMI). Patients were diagnosed with MINOCA if they met the 4^th^ universal definition of AMI (23) and the coronary angiography did not show a stenosis of ≥50% in epicardial coronary arteries (2). Patients were excluded due to: (1) presence of obstructive CAD (n=21696); (2) prior revascularization (n=312); (3) thrombolytic therapy for STEMI since the coronary lesion may be affected by thrombolysis (n=126); (4) alternate explanations for elevated troponin rather than coronary-related causes (e.g., acute heart failure, myocarditis, pulmonary embolism, takotsubo syndrome, n=46); (5) concomitant with hyperthyroidism (n=7) or hypothyroidism (n=10); (6) lack of detailed baseline data (n=33); (7) lost at follow up (n=68). As a result, 1162 eligible MINOCA patients with euthyroid were enrolled in final analysis (**Figure 1**). Patients were prescribed the evidence-based secondary therapies, including dual anti-platelet therapy (DAPT), statins, β-blocker, and angiotensin-converting enzyme inhibitor (ACEI) or angiotensin receptor antagonist (ARB) (24, 25). This study was approved by the Ethics Committee of Fuwai hospital and complied with the Declaration of Helsinki. All enrolled subjects provided the written informed consent.

### Data Collection

Patients’ demographics, medical history, laboratory test, echocardiographic data and medication were collected and verified from in-person interviews and medical records. Body mass index (BMI) was calculated as weight (kg) divided by height (m) squared. The thyroid function profiles including fT3, fT4, and TSH were measured using a direct chemiluminescence method (ADVIA Centaur, Siemens, USA). The reference intervals were as follows: fT3, 2.36-4.21 pg/mL; fT4, 0.89-1.76 ng/dL; TSH, 0.55-4.78 μIU/mL. The concentrations of fasting blood glucose (FBG), low density lipoprotein cholesterol (LDL-C), creatinine, and high-sensitive C-reactive protein (hs-CRP) were tested using an automatic biochemistry analyzer. The N-terminal po-B-type natriuretic peptide (NT-proBNP) and cardiac troponin I (TnI) values at admission were recorded. The left ventricular ejection fraction (LVEF) was measured using echocardiography with the biplane Simpson method. The Thrombolysis in Myocardial Infarction (TIMI) risk score was calculated since admission as previously described (26, 27).

### Definitions and Outcomes

In this study, euthyroidism was defined as having no history of hyperthyroidism or hypothyroidism and with normal levels of fT3, fT4, and TSH (11). Diabetes was defined with FBG ≥7.0 mmol/L, 2-h plasma glucose ≥11.1 mmol/L, or having a diabetic history (28). Hypertension was defined as repeated blood pressure ≥140/90 mmHg, use of anti-hypertensive drugs, or having a history of hypertension. Dyslipidemia was diagnosed by medical history or having LDL-C≥3.4 mmol/L, high density lipoprotein cholesterol <1.0 mmol/L, or triglyceride≥ 1.7mmol/L (29).

The primary study endpoint was a composite of major adverse cardiovascular events (MACE), including all-cause death, nonfatal MI, revascularization, nonfatal stroke, and hospitalization for unstable angina (UA) or heart failure (HF). The MACE was assessed as time to first event. The secondary endpoints included each component of MACE and the composite “hard” endpoint of death, nonfatal MI, revascularization, and nonfatal stroke. Reinfarction was diagnosed according to the 4^th^ universal definition of MI (23). Revascularization was performed at the operator’s discretion due to recurrent ischemia and progression of coronary lesion. Stroke was defined by the presence of neurological dysfunction and vascular brain injury caused by cerebral ischemia or hemorrhage (30). Hospitalization for UA or HF reflected the clinical status and quality of life after AMI. Patients were regularly followed up at clinics or *via* telephone by a team of independent researchers. The endpoints were confirmed by at least two professional cardiologists.

### Statistical Analysis

Data were expressed as mean ± standard deviation (SD) or median with interquartile range for continuous variables and numbers with percentages for categorical variables. Differences were evaluated using the analysis of variance or Kruskal-Wallis H test for continuous variables and Pearson’s χ^2^ or Fisher’s exact test for categorical variables. Cumulative incidence of MACE among groups were showed by Kaplan-Meier analysis and compared using log-rank test. The univariable and multivariable Cox proportional regression analyses were used to identify association between levels of fT3/fT4 ratio and outcomes. The risk of MACE was adjusted by age and sex and further adjusted by multiple clinically relevant variables, including age, sex, MI classification (NSTEMI or STEMI), hypertension, diabetes and dyslipidemia. The hazard ratio (HR) with 95% confidence interval (CI) were calculated. Discrimination was defined with areas under the curve (AUC) using a receiver-operating characteristic curve (ROC) analysis. The AUC values were categorized as negligible (≤0.55), small (0.56-0.63), moderate (0.64-0.70) or strong (≥0.71) (31), and compared by Delong’s test (32) with MedCalc version 11.4 (MedCalc Inc., Ostend, Belgium). A combined risk model incorporating fT3/fT4 ratio into the original TIMI risk score was generated using Cox regression. A two-tailed P<0.05 was considered statistically significant. Unless stated otherwise, most of the analyses were performed with SPSS version 22.0 (SPSS Inc., Chicago, IL, USA).

## Results

### Baseline Characteristics

Patients were divided according to decreasing tertile levels of fT3/fT4 ratio (tertile1: fT3/fT4 ≥2.64, n=387; tertile2: 2.28≤ fT3/fT4 <2.64, n=386; tertile3: fT3/fT4 <2.28, n=389) (**Figure 1**). As shown in **Table 1**, patients with lower fT3/fT4 tertiles were older and more often female. They had more presence of STEMI and higher prevalence of hypertension and diabetes. They also had higher Killip class, lower LVEF, higher TIMI score, and higher values of FBG, hs-CRP, NT-proBNP and peak TnI. There were no significant differences in BMI, dyslipidemia, prior MI, LDL-C, creatinine levels, and in-hospital medication among the 3 groups. In this regard, patients with lower levels of fT3/fT4 ratio had more baseline risk profiles.

**Table 1:** Baseline characteristics in MINOCA patients based on tertiles of fT3/fT4 ratio.

Variable	Tertile1 (n=387)	Tertile2 (n=386)	Tertile3 (n=389)	p value
Male, n(%)	300 (77.5%)	291 (75.3%)	265 (68.1%)	<0.001
Age, years	54.0±12.3	55.1±11.6	57.8±11.1	<0.001
BMI, kg/m^2^	25.2±3.5	25.4±3.8	25.8±3.9	0.252
STEMI, n(%)	128 (33.0%)	155 (40.1%)	173 (44.4%)	0.022
Past history				
Hypertension	195 (50.3%)	204 (52.8%)	220 (56.5%)	0.035
Diabetes	51 (13.1%)	57 (14.7%)	75 (19.2%)	0.043
Dyslipidemia	231 (59.6%)	225 (58.2%)	217 (55.7%)	0.172
Previous MI	18 (4.6%)	20 (5.1%)	20 (5.1%)	0.931
Killip class≥2, n(%)	21 (5.4%)	29 (7.5%)	36 (9.2%)	0.036
LVEF, %	61.2±6.0	60.9±6.0	59.4±7.5	<0.001
TIMI risk score	3.2±1.1	3.4±1.2	3.7±1.5	0.002
Laboratory test				
fT3, pg/mL	3.03±0.36	2.87±0.31	2.56±0.39	<0.001
fT4, ng/dL	1.04±0.13	1.17±0.13	1.28±0.19	<0.001
fT3/fT4 ratio	2.93±0.27	2.46±0.10	2.01±0.21	<0.001
TSH, μIU/mL	2.13±1.51	2.14±1.59	2.08±1.33	0.162
FBG, mmol/L	5.53±1.55	5.60±1.51	5.95±1.92	0.001
LDL-C, mmol/L	2.28±0.78	2.30±0.73	2.29±0.76	0.901
Creatinine, μmol/L	78.9±14.5	79.9±15.2	81.4±18.7	0.566
hs-CRP, mg/L	1.98 (0.95, 4.40)	2.09 (1.07, 5.17)	2.86 (1.17, 8.40)	<0.001
NT-proBNP, pg/mL	302 (107, 653)	351 (118, 695)	403 (121, 786)	0.023
TnI, ng/mL	1.31 (0.32, 3.73)	1.52 (0.41, 4.85)	1.76 (0.72, 5.13)	0.011
In-hospital medication				
DAPT	353 (91.2%)	364 (94.3%)	361 (92.8%)	0.253
Statin	372 (96.1%)	368 (95.3%)	373 (95.8%)	0.856
ACEI or ARB	243 (62.7%)	238 (61.6%)	263 (67.6%)	0.186
Beta-blocker	269 (69.5%)	284 (73.5%)	295 (75.8%)	0.133

Patients were divided according to decreasing tertile levels of fT3/fT4 ratio (Tertile1: fT3/fT4≥2.64, Tertile2: 2.28≤ fT3/fT4 <2.64, Tertile3: fT3/fT4 <2.28). BMI, body mass index; STEMI, ST-segment elevation myocardial infarction; LVEF, left ventricular ejection fraction; TIMI, Thrombolysis in Myocardial Infarction; fT3, free triiodothyronine; fT4, free thyroxine; TSH, thyroid-stimulating hormone; FBG, fasting blood glucose; LDL-C, low-density lipoprotein cholesterol; hs-CRP, high-sensitive C-reactive protein; NT-proBNP, N-terminal pro-B-type natriuretic peptide; TnI, Troponin I; DAPT, dual anti-platelet therapy; ACEI, angiotensin-converting enzyme inhibitor; ARB, angiotensin receptor antagonist.

### Association Between fT3/fT4 Ratio and Outcomes

**Figure 2:**
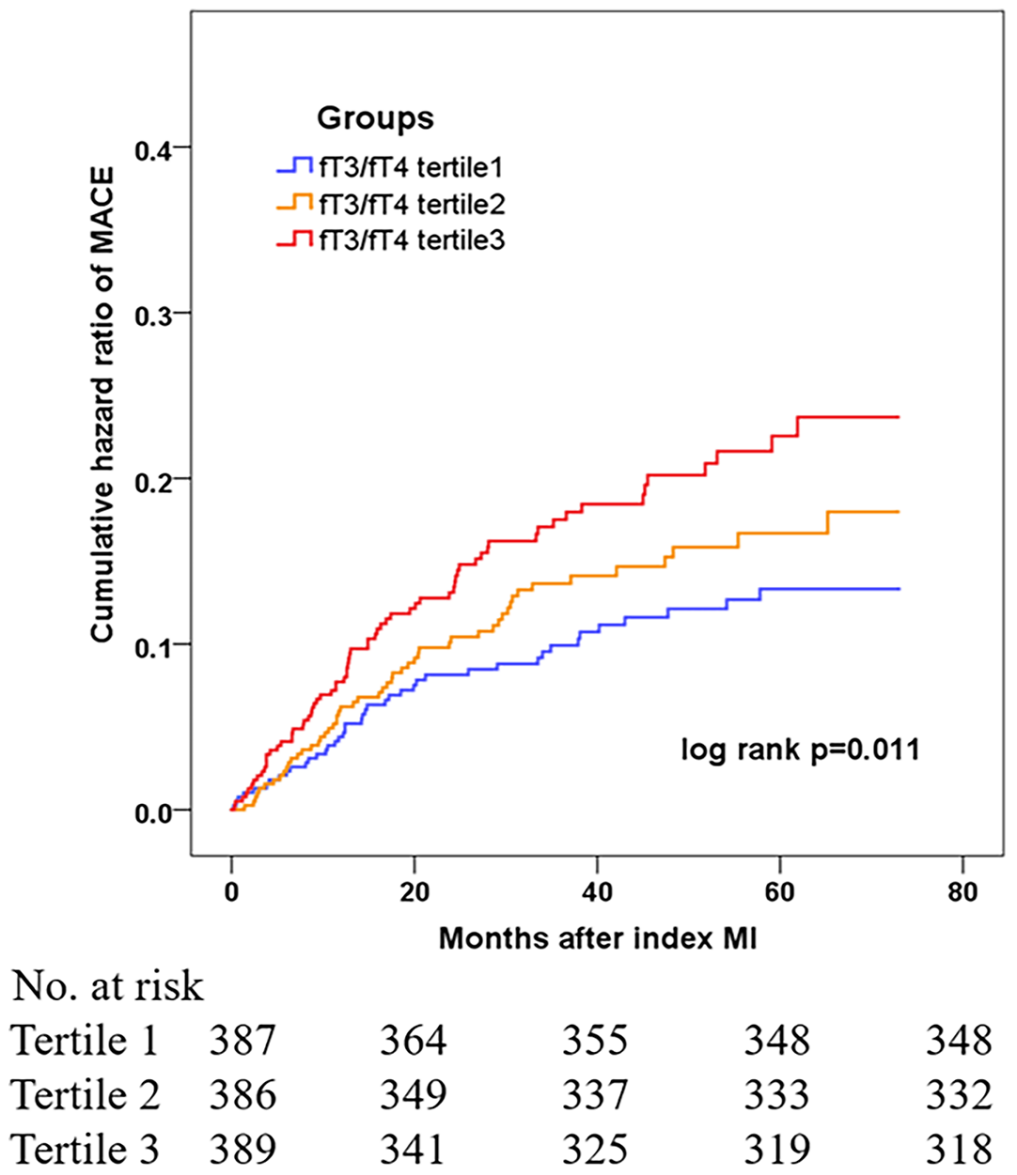
Cumulative hazard ratio of MACE in MINOCA patients based on tertile levels of fT3/fT4 ratio.

During the median follow-up of 41.7 months, 164 euthyroid patients with MINOCA developed MACE (16 died, 40 had recurrent MI, 46 had revascularization, 12 suffered stroke, 70 was hospitalized for UA and 46 hospitalized for HF) (**Table 2**). Patients with lower fT3/fT4 had a significantly higher incidence of MACE (10.0%, 13.9%, 18.2%; p=0.005). The rate of the composite endpoint of death, reinfarction, revascularization, or stroke also increased with decreasing fT3/fT4 tertiles (5.4%, 8.5%, 11.5%; p=0.035). In addition, the Kaplan-Meier analysis showed that the cumulative incidence of MACE was significantly higher in patients with lower fT3/fT4 (log rank p=0.011) (**Figure 2**).

**Table 2:** Clinical outcomes in MINOCA patients based on tertiles of fT3/fT4 ratio.

CV outcomes	Tertile1 (n=387)	Tertile2 (n=386)	Tertile3 (n=389)	p value
MACE	39 (10.0%)	54 (13.9%)	71 (18.2%)	0.005
Death, nonfatal MI, stroke or revascularization	21 (5.4%)	33 (8.5%)	45 (11.5%)	0.035
All-cause death	3 (0.7%)	3 (0.7%)	10 (2.5%)	0.047
Nonfatal MI	9 (2.3%)	12 (3.1%)	19 (4.8%)	0.072
Revascularization	7 (1.8%)	18 (4.6%)	21 (5.3%)	0.031
Nonfatal stroke	4 (1.0%)	3 (0.7%)	5 (1.2%)	0.359
Hospitalization for UA	14 (3.6%)	24 (6.2%)	32 (8.2%)	0.018
Hospitalization for HF	12 (3.1%)	10 (2.6%)	24 (6.1%)	0.022

Tertile1: fT3/fT4≥2.64, Tertile2: 2.28≤ fT3/fT4 <2.64, Tertile3: fT3/fT4 <2.28. MACE, major adverse cardiovascular events; MI, myocardial infarction; UA, unstable angina; HF, heart failure.

**Figure 3:**
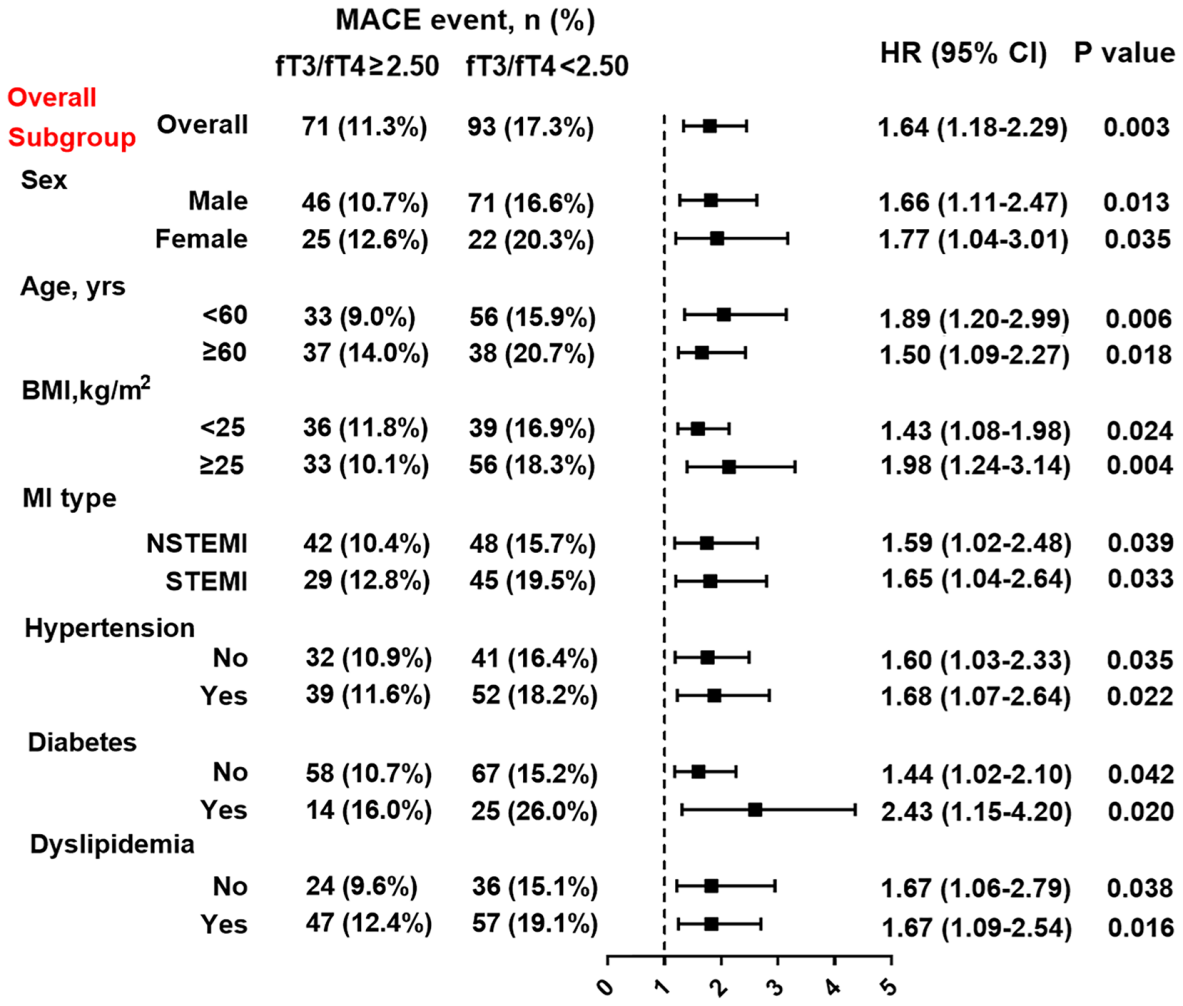
Association between fT3/fT4 ratio and the risk of MACE in overall and subgroups. Subgroup analysis showing the incidence and risk of MACE in patients with fT3/fT4 ratio above and below the cut-off value of 2.50, which was identified with maximum Youden index in all MINOCA patients for MACE prediction. Hazard ratio (HR) was calculated by univariate Cox regression analysis. Vertical dotted line indicated the HR value of 1. BMI, body mass index; STEMI, ST-segment elevation myocardial infarction; NSTEMI; non-ST-segment elevation myocardial infarction, CI, confidence interval.

At multivariate Cox analysis, patients with lower fT3/fT4 tertiles had an increased risk of MACE after adjustment for age and sex (tertile 1 as reference; tertile 2: HR 1.69, 95% CI: 1.11-2.56, p=0.014; tertile 3: HR 2.17 95% CI: 1.25-3.26, p=0.001) or after multivariate adjustment (tertile 1 as reference; tertile 2: HR 1.58, 95% CI: 1.05-2.39, p=0.030; tertile 3: HR 2.06, 95% CI: 1.17-3.11, p=0.006) (**Table 3**). Meanwhile, the fT3/fT4 level was significantly correlated with the adjusted risk of MACE (for per 1SD increase in fT3/fT4, HR 0.57, 95% CI: 0.38-0.84, p=0.005) (**Table 3**). At ROC analysis, the cutoff of fT3/fT4 that maximized sensitivity and specificity for MACE prediction in all patients was identified as 2.50. Totally, 535 patients (46.0%) had a ratio above the cutoff value. The incidence of MACE was 11.3% and 17.3% (p<0.001) in patients with fT3/fT4 above and below the cutoff, respectively (HR 1.64, 95% CI: 1.18-2.29, p=0.003) (**Figure 3**). At subgroup analysis, lower fT3/fT4 (<2.50) remained a robust risk factor in subsets of patients stratified by sex, age, BMI, MI classification, history of hypertension, diabetes, and dyslipidemia (all p<0.05) (**Figure 3**), suggesting that the prognostic effect of fT3/fT4 was not affected by clinically relevant demographic or traditional risk factors.

**Table 3:** Association between tertile levels of fT3/fT4 ratio and the risk of MACE.

Group	Unadjusted		Model 1		Model 2	
	HR (95% CI)	P value	HR (95% CI)	P value	HR (95% CI)	P value
fT3/fT4 ratio, per 1SD increase	0.49 (0.33-0.71)	<0.001	0.53 (0.36-0.77)	0.001	0.57 (0.38-0.84)	0.005
Tertile 1	1 (reference)	…	1 (reference)	…	1 (reference)	…
Tertile 2	1.71 (1.12-2.61)	0.013	1.69 (1.11-2.56)	0.014	1.58 (1.05-2.39)	0.030
Tertile 3	2.22 (1.28-3.33)	<0.001	2.17 (1.25-3.26)	0.001	2.06 (1.17-3.11)	0.006

Tertile1: fT3/fT4≥2.64, Tertile2: 2.28≤ fT3/fT4 <2.64, Tertile3: fT3/fT4 <2.28. Model 1 included age and sex. Model 2 included age, sex, MI classification (NSTEM or STEMI), hypertension, diabetes and dyslipidemia in the multivariate Cox analysis. fT3, free triiodothyronine; fT4, free thyroxine; HR, hazard ratio; CI, confidence interval; SD, standard deviation.

### Predictive Value of fT3/fT4 Ratio for MACE

**Figure 4:**
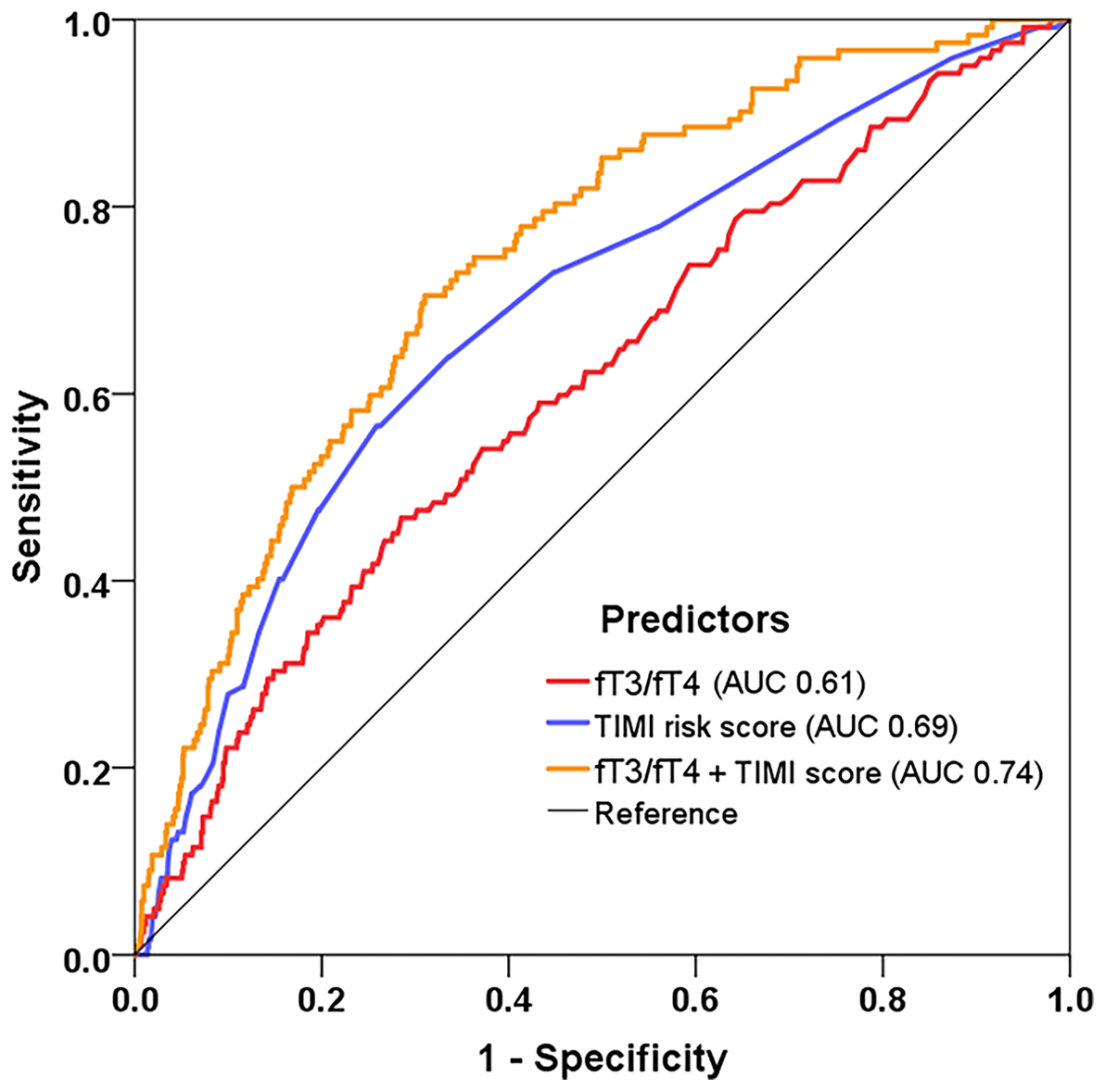
Model improvement in predicting MACE. Receiver operating characteristic curves showing predictive value of the fT3/fT4 ratio, TIMI risk score, and the combined model incorporating fT3/fT4 and TIMI score using Cox regression. fT3, free triiodothyronine; fT4, free thyroxine; TIMI, Thrombolysis in Myocardial Infarction.

The ROC analysis confirmed the value of fT3/fT4 ratio for MACE prediction (AUC 0.61, 95% CI: 0.55-0.66, p<0.001) (**Figure 4**). Meanwhile, the TIMI risk score showed a moderate discrimination for MACE (AUC 0.69, 95% CI: 0.64-0.73, p<0.001). When adding fT3/fT4 ratio to the original TIMI risk score using Cox regression, the combined model yielded a significant improvement in risk prediction (AUC increased from 0.69 to 0.74, ΔAUC 0.05, p=0.023 by DeLong’s test).

## Discussion

The present study, for the first time, verified the clinical significance of the fT3/fT4 ratio in euthyroid patients with MINOCA, and found that decreased fT3/fT4 ratio was independently associated with an increased risk of long-term MACE. Adding fT3/fT4 ratio to traditional risk score further improved the outcome prediction. Our data support the utility of fT3/fT4 ratio as a prognostic marker for risk stratification in contemporary real-world management of MINOCA.

MINOCA represents a distinct clinical entity with multiple underlying mechanisms, including plaque rupture or erosion, thromboembolism, spasm, spontaneous dissection, microvascular dysfunction and supply/demand mismatch. Some non-ischemic diseases such as myocarditis may also mimic the presentation of MINOCA (5). More recently, however, it has been used to primarily describe patients with coronary-related ischemia. We adopted this criteria and established a long-term cohort with relatively large sample size. A systematic review estimated the prevalence of MINOCA to be 6% in all AMIs (4), which is close to the prevalence of 5.1% in our study. As reported, nearly one-third of MINOCA would present with STEMI. Patients with MINOCA were more likely to be younger, female, and had fewer comorbidities compared to patients with AMI and obstructive CAD (4). We described the clinical profiles of MINOCA across the fT3/fT4 ratio tertile levels. Meanwhile, we found that the course of MINOCA was not benign. Over the median follow-up of 3.5 years, about 1.4% of MINOCA patients died and 14.1% of them developed MACE. Similarly, previous studies showed that these patients were still at considerable risks for long-term mortality and CV events after MINOCA (4–10). Therefore, the prognosis of MINOCA should be more emphasized and it is necessary to find potential residual risk factors and further improve healthcare for this population.

Thyroid function has a complex relationship with CV physiology (11, 12). Overt thyroid disease or even subclinical thyroid dysfunction can result in alterations in cardiac output, heart rate, vascular resistance, and blood pressure (11). There are also significant changes in atherosclerotic risk factors, including dyslipidemia, hypertension, hypercoagulation, arterial stiffness, and LV dysfunction (12), which may markedly increase CV morbidity and mortality. In addition, euthyroid patients with CV disease may also have alterations in TH concentrations that are associated with worse outcomes (13, 14). TH mainly consist of iodinated T3 and T4 and the free forms of fT3 and fT4. Previous studies have verified the prognostic values of free TH in euthyroid patients with CAD. Lower level of fT3 is commonly seen in AMI, which is not only associated with the severity of myocardial injury and LV dysfunction, but also predicts poor prognosis after AMI (33–35). Meanwhile, high fT4 level is also a potential CV risk factor. Several population-based studies have confirmed that higher fT4 level is independently correlated with atherosclerosis (36) and increased risks of MACE even in euthyroid subjects (37–39). Recent evidence suggest that the combined evaluation of fT3 and fT4 (fT3/fT4 ratio) may serve as a reasonable index of metabolic variation of TH compared with fT3 or fT4 alone and may thus provide a more accurate outcome prediction in different clinical settings. A British cohort study found that lower fT3/fT4 ratio was associated with frailty and long-term mortality in hospitalized older patients (17). The fT3/fT4 ratio was still a robust risk factor for cardiac dysfunction and 1-year mortality in dilated cardiomyopathy (18). In terms of CAD, the fT3/fT4 ratio was reported to be inversely associated with an increased risk of death in euthyroid patients with ACS (19–21). This is not only the case in acute setting, but also in the longer term after recovery from ACS. Another study also indicated that low fT3/fT4 ratio was related to long-term MACE in euthyroid patients with three-vessel disease (22).

In line with previous results, we found that the incidence and adjusted risk of MACE significantly increased with the decreasing tertiles of fT3/fT4 ratio. The fT3/fT4 ratio was inversely correlated with risk of MACE. Meanwhile, lower fT3/fT4 ratio defined by the cut-off of 2.50 remained an independent predictor of MACE in overall and in subgroups. Further, fT3/fT4 ratio provided an incremental predictive value of MACE when added to the TIMI risk score. These results extended the utility of fT3/fT4 ratio to euthyroid patients with MINOCA, suggesting that it might be reasonable to use the ratio as a prognostic marker in daily clinical practice for this specific population.

Actually, both T3 and T4 constitute the active forms of TH, but the majority of T3 is converted from T4 in the process of peripheral deiodination. Moreover, T3 has a higher affinity than T4 for TH receptors in myocardium and vascular tissue, and exerts various bioactive effects on CV system *via* nongenomic and genomic approaches (40). Thus, the conversion of T4 to T3 is critical for the production of circulating T3 and the TH actions on CV function. Decrease in fT3/fT4 ratio reflects the disturbance of T4 converting to T3, and is commonly seen in acute or chronic processes of myocardial injury as cardiac disease itself may lead to alterations in TH concentrations (17–19). Lower fT3/fT4 ratio, in turn, have deleterious effects on CV systems including reduced cardiac contractility and increased vascular resistance (12–14). Other mechanisms such as cardiac dysfunction, inflammation and oxidative stress have also been proposed (41, 42). Several studies, along with ours, have found that CAD patients with lower fT3/fT4 ratio tended to have more severe myocardial injury (e.g., higher TnI), more impaired LV mechanics (e.g., lower LVEF, higher NT-proBNP) and higher plasma inflammatory markers (e.g., higher hs-CRP) (20–22). All these changes may finally contribute to the increased risks of CV events. However, the pathophysiological and therapeutic relevance of thyroid dysregulation in euthyroid patients after AMI are far from elucidated. Future studies are warranted to confirm our findings and to better understand the biological mechanisms underlying this prognostic association.

## Limitation

Some limitations should be mentioned. First, the percentage of women was relatively low in our cohort, possibly due to the large proportion of men in all AMIs treated in our center and a lower rate for women to receive coronary angiography. Given the potential selection bias in single-center studies, future nationwide registry cohorts of MINOCA are warranted to validated our findings. Second, we did not capture and record the exact mechanism for every MINOCA patient. The association between etiology of MINOCA and outcomes should be further investigated. Third, despite multivariate adjustment and subgroup analyses were performed, there might be other unmeasured confounders that may affect the prognosis. Fourth, the fT3/fT4 ratio was only measured at baseline, and the follow-up levels of fT3/fT4 ratio may also be clinically significant.

## Conclusion

Decreased fT3/fT4 ratio was an independent predictor of poor outcomes in euthyroid patients with MINOCA. Routine assessment of fT3/fT4 ratio might provide significant prognostic value and facilitate risk stratification and decision making in this population.

## Data Availability Statement

The raw data supporting the conclusions of this article will be made available by the authors, without undue reservation.

## Ethics Statement

The studies involving human participants were reviewed and approved by Ethics Committee of Fuwai hospital. The patients/participants provided their written informed consent to participate in this study.

## Author Contributions

SG conceived and designed the study. SG, WM, SH and XL performed data analysis and interpretation. SG drafted the manuscript. MY reviewed and gave final approval of the version to be published. All authors contributed to the article and approved the submitted version.

## Funding

This work was supported by National Natural Science Foundation of China (81670415).

## Conflict of Interest

The authors declare that the research was conducted in the absence of any commercial or financial relationships that could be construed as a potential conflict of interest.

## Publisher’s Note

All claims expressed in this article are solely those of the authors and do not necessarily represent those of their affiliated organizations, or those of the publisher, the editors and the reviewers. Any product that may be evaluated in this article, or claim that may be made by its manufacturer, is not guaranteed or endorsed by the publisher.
