# Supplementary material for: Predictive Value of Free Triiodothyronine to Free Thyroxine Ratio in Euthyroid Patients With Myocardial Infarction With Nonobstructive Coronary Arteries
Source: Front Endocrinol (Lausanne). 2021 Jul 28;12:708216. doi: 10.3389/fendo.2021.708216 (PMC8356082; doi:10.3389/fendo.2021.708216)
Supplement: Supplementary file 1 [file Table_1.docx]

**Supplementary data of the manuscript (for reviewer)**

**Suppl Table. Relationship between thyroid hormone levels and the risk of MACE.**

| Variable | Unadjusted Cox analysis | | Adjusted Cox analysis | |
| --- | --- | --- | --- | --- |
|  | HR (95% CI) | P value | HR (95% CI) | P value |
| fT4 | 2.45 (1.10-5.43) | 0.027 | 2.23 (0.92-5.41) | 0.075 |
| fT3 | 0.58 (0.40-0.85) | 0.006 | 0.69 (0.47-1.01) | 0.054 |
| TSH | 0.96 (0.85-1.07) | 0.499 | 0.95 (0.85-1.07) | 0.387 |
| fT3/fT4 | 0.49 (0.33-0.71) | <0.001 | 0.57 (0.38-0.84) | 0.005 |

Hazard ratio (HR) was expressed as per 1 SD increase in thyroid hormones. Age, sex, MI type (NSTEM or STEMI), hypertension, diabetes, and dyslipidemia were enrolled in the multivariate model. fT3: free triiodothyronine, fT4: free thyroxine, TSH: thyroid-stimulating hormone, CI: confidence interval.
